# Psychological Interventions for Children with Neurodevelopmental Disabilities in the Context of Neurodiversity: A Systematic Review

**DOI:** 10.3390/children13081071

**Published:** 2026-08-12

**Authors:** Kalliopi Megari, Dimitra V. Katsarou, Evangelos Mantsos, Soultana Papadopoulou, Efthymia Efthymiou, Maria Theodoratou, Maria Sofologi, Eugenia Toki

**Affiliations:** 1 Department of Psychology-CITY ULE, University of York, Europe Campus, 54626 Thessaloniki, Greece; 2 School of Social Sciences and Humanities, Department of Psychology, University of Western North Macedonia, 53100 Florina, Greece; 3 Department of Preschool Education Sciences and Educational Design, University of the Aegean, 85132 Rhodes, Greece; d.katsarou@aegean.gr; 4 Department of Occupational Therapy, University of Western Macedonia, 50200 Ptolemaida, Greece; 5 Department of Speech Therapy, School of Health Sciences, University of Ioannina, 45500 Ioannina, Greece; papasoul@uoi.gr (S.P.);; 6 College of Interdisciplinary Studies, Zayed University, Dubai 144534, United Arab Emirates; efthymia.efthymiou@zu.ac.ae; 7 Department of Psychology, School of Health Sciences, Neapolis University, Pafos 8042, Cyprus; m.theodoratou@nup.ac.cy; 8 School of Social Sciences, Hellenic Open University, 26335 Patra, Greece; 9 Department of Early Childhood Education, School of Education, University of Ioannina, 45110 Ioannina, Greece; m.sofologi@uoi.gr

**Keywords:** neurodiversity, psychological interventions, autism spectrum disorder, learning disabilities, children, quality of life

## Abstract

**Highlights:**

**What are the main findings?**

- From a neurodiversity viewpoint, this systematic review summarized research on psychological therapies for children with neurodevelopmental disorders.
- Conventional therapies like Cognitive Behavioral Therapy (CBT) and Applied Behavior Analysis (ABA) have been shown in 34 studies to enhance cognitive functioning, emotional control, and adaptive skills.

**What is the implication of the main finding?**

- The results encourage a shift toward tailored, strengths-based, and context-responsive therapies that acknowledge neurodivergent variations as a normal aspect of human variability rather than as deficiencies that need to be fixed.
- More varied neurodevelopmental populations should be included in future studies, better longitudinal designs should be used, and neurodivergent people should be actively included in the creation and assessment of therapies aimed at enhancing long-term functioning, involvement, and quality of life.

**Abstract:**

Background/Objectives: This systematic review examines psychological interventions for children with neurodevelopmental disabilities within a neurodiversity framework, emphasizing that variations in cognition, behavior, and neurological functioning represent natural forms of human diversity rather than deficits. Methods: A systematic literature search was conducted across the PubMed, PsycINFO, and Google Scholar databases for studies published between 2010 and 2025. Studies were included if they focused on children with autism spectrum disorder (ASD), learning or intellectual disabilities (LD/ID), or specific learning disabilities (SpLD), and evaluated psychological or behavioral interventions. Results: A total of 34 studies met the inclusion criteria after screening and full-text review. The findings indicate that traditional interventions, such as Applied Behavior Analysis (ABA) and Cognitive Behavioral Therapy (CBT), demonstrate effectiveness in improving adaptive behavior, emotional regulation, and cognitive functioning. However, more recent approaches, including Naturalistic Developmental Behavioral Interventions (NDBI), person-centered therapies, and gamified interventions, show greater alignment with neurodiversity principles by emphasizing individual strengths, autonomy, and environmental adaptation. Conclusions: Despite promising outcomes, the evidence base remains uneven, with a predominant focus on autism and limited high-quality studies addressing other neurodevelopmental conditions. The review highlights the need for more rigorous, inclusive, and longitudinal research, as well as the integration of neurodivergent perspectives in intervention design. Overall, the findings suggest that interventions incorporating individualized, strengths-based, and context-sensitive adaptations are associated with more consistent improvements in both functional outcomes and quality of life.

## 1. Introduction

The concept of neurodiversity has emerged as a paradigm shift in understanding neurodevelopmental differences, emphasizing that variations in cognition, behavior, and neurological functioning represent natural forms of human diversity rather than deficits [1,2,3]. Recent perspectives further emphasize that cognitive diversity can drive innovation and adaptive functioning across contexts [4]. Within this framework, conditions such as autism spectrum disorder (ASD), learning and intellectual disabilities (LD/ID), and specific learning disabilities (SpLD) are increasingly viewed through a strengths-based and person-centered lens [5]. This perspective challenges traditional deficit-oriented models and calls for approaches that recognize individual differences, promote inclusion, and enhance quality of life.

It is important to distinguish between neurodiversity as a conceptual framework and neurodevelopmental disabilities as clinical diagnostic categories. In this review, the term *neurodivergent children* refers to children whose developmental trajectories differ from neurotypical patterns, including those diagnosed with ASD, LD/ID, and SpLD. Neurodevelopmental disabilities are formally classified within diagnostic frameworks such as the *Diagnostic and Statistical Manual of Mental Disorders* [6], which provide standardized criteria for identifying developmental differences. These children often present with diverse cognitive profiles, encompassing differences in attention, memory, executive functioning, and social communication [7,8], which necessitate tailored and flexible intervention approaches. Early identification and intervention are critical, as developmental trajectories in autism and related conditions are highly sensitive to timing and intensity of support [9].

Traditional psychological interventions have historically focused on symptom reduction and deficit remediation, particularly in the context of autism and learning disabilities. Approaches such as Applied Behavior Analysis (ABA) and Cognitive Behavioral Therapy (CBT) have demonstrated effectiveness in improving specific functional outcomes, including communication, behavior regulation, and academic performance [10,11]. However, these approaches have also been critiqued for emphasizing normalization and overlooking the lived experiences and autonomy of neurodivergent individuals [12,13].

In response to these concerns, there has been a growing shift toward neurodiversity-affirming practices, which prioritize individual strengths, environmental adaptation, and meaningful participation [14]. Emerging intervention models, such as Naturalistic Developmental Behavioral Interventions (NDBIs), person-centered therapies, and technology-enhanced approaches (e.g., gamification), aim to support development while respecting neurodivergent identities [15,16]. These approaches align with broader frameworks of inclusion, self-determination, and well-being. A modern class of evidence-based therapy known as Naturalistic Developmental Behavioral therapy (NDBIs) combines applied behavior analysis and developmental psychology concepts in naturalistic, child-led settings. NDBIs place more emphasis on learning through play, ordinary interactions, and meaningful social experiences than typical highly regimented behavioral techniques. Therapists and caregivers are encouraged by interventions like the Early Start Denver Model (ESDM), Joint Attention Symbolic Play Engagement and Regulation (JASPER), and Pivotal Response Treatment (PRT) to follow the child’s interests while incorporating educational opportunities into everyday routines. Responsive communication, shared engagement, setting up the environment, modeling, natural reinforcement, and active caregiver participation are all essential elements. NDBIs seek to foster more general developmental abilities, such as social communication, language acquisition, emotional control, adaptive functioning, and reciprocal interaction, rather than focusing on specific behaviors [15,16].

The importance of person-centered therapeutic approaches in providing children with neurodevelopmental challenges with psychological care that affirms their neurodiversity is becoming more widely acknowledged. These interventions, which have their roots in humanistic psychology, place a strong emphasis on empathy, unconditional positive regard, cooperative goal-setting, and respect for the individual strengths, preferences, and life experiences of each child. Person-centered therapies emphasize self-understanding, emotional well-being, self-advocacy, resilience, and meaningful engagement in family, school, and community life rather than seeing neurodevelopmental variations as weaknesses that need to be corrected. Individualized psychotherapy modifications, family-centered therapies, strengths-based coaching, and collaborative care planning—which actively involve children and caregivers in treatment decisions—are frequently used in clinical practice. This method recognizes that psychological suffering often results from contextual obstacles, stigma, sensory overload, and insufficient accommodations in addition to neurodevelopmental traits.

The variety of psychological interventions for children with neurodevelopmental challenges has significantly increased because of technological developments. Telehealth-delivered psychotherapy, mobile health apps, virtual reality (VR), augmented reality (AR), serious games, gamified cognitive training, wearable technology, AI-supported adaptive learning platforms, and digital therapeutics are all examples of technology-enhanced interventions. Increased accessibility for families in underserved areas, chances for intense and repeated practice, performance-based individualized progression, real-time feedback, and increased engagement through interactive multimedia environments are just a few of the useful benefits that these approaches offer. Virtual reality has shown potential in enhancing executive functioning, social communication, emotional awareness, and adaptive skills by giving kids safe, realistic environments to practice commonplace scenarios. Gamified interventions target cognitive, behavioral, and social goals while increasing engagement by utilizing motivating concepts. By lowering logistical and geographic barriers to care, telehealth has further enabled the delivery of interdisciplinary interventions and caregiver coaching. Crucially, rather than enforcing uniform behavioral expectations, many digital therapies are being created in accordance with neurodiversity principles by permitting customized sensory experiences, flexible pacing, adaptable interfaces, and unique therapeutic goals.

Despite the increasing interest in neurodiversity-informed practices, the evidence base remains fragmented, with a strong emphasis on autism and limited integration across different neurodevelopmental conditions. Furthermore, there is a need to critically examine how existing psychological interventions align with neurodiversity principles and to identify gaps in current research.

Therefore, in the context of neurodiversity, this systematic review sought to compile data from original empirical studies assessing psychological therapies for children with neurodevelopmental impairments. In order to inform future clinical practice and research, the review specifically aimed to identify current intervention approaches; assess their efficacy in enhancing cognitive, behavioral, emotional, social, and adaptive outcomes; assess the degree to which interventions reflect neurodiversity-affirming principles; and identify current evidence gaps. Specifically, the review seeks to (a) outline emerging approaches in interventions used as gamification across diagnostic groups, (b) evaluate their effectiveness, and (c) examine the extent to which these interventions align with neurodiversity-affirming principles.

## 2. Materials and Methods

### 2.1. Study Design

This systematic review was conducted in accordance with the Preferred Reporting Items for Systematic Reviews and Meta-Analyses (PRISMA) guidelines to ensure transparency and methodological rigor in the identification, selection, and synthesis of relevant studies.

The review protocol was prospectively registered in the PROSPERO database (International Prospective Register of Systematic Reviews) (Registration No. CRD420261406458.), ensuring adherence to predefined methodological procedures. PROSPERO, is an international database for the registration of systematic review protocols. Prospective registration offers a publicly available record of the intended review approach, encourages transparency, and lowers the possibility of selective reporting.

### 2.2. Search Strategy

A systematic literature search was conducted across the electronic databases PubMed, PsycINFO, and Google Scholar to identify relevant studies including clinical studies. The search covered publications from January 2010 to December 2025, reflecting contemporary developments in neurodiversity-informed practices (Table 1 and Table 2).

### 2.3. Study Selection Process

The study selection process was conducted in two stages. First, titles and abstracts were screened for relevance. Second, full-text articles were assessed against the inclusion criteria.

A total of 128 records were initially identified through database searching. After the removal of 32 duplicates, 96 records remained for screening. Following title and abstract screening, 58 articles were excluded due to irrelevance or failure to meet inclusion criteria.

The full texts of 38 studies were assessed for eligibility, of which 4 were excluded due to insufficient methodological detail or lack of intervention outcomes.

Ultimately, 34 studies were included in the final analysis.

Two authors (K.M. & D.V.K.) independently selected the studies in two steps: (1) screening of abstracts and titles and (2) evaluation of full-text eligibility. All obtained records were independently assessed by both reviewers using the predetermined inclusion and exclusion criteria. Discussion and agreement were used to settle any disagreements on the choice of studies. During the selection procedure, there was no official scoring of the research.

### 2.4. Quality Assessment

The methodological quality of included studies was assessed using the Cochrane Risk of Bias tool for randomized controlled trials and the Newcastle–Ottawa Scale for observational studies.

Overall, the quality of the included studies ranged from moderate to high, although variability was observed in sample size, intervention duration, and outcome measurement.

### 2.5. Data Synthesis

We replied to the research questions with findings that were grouped into thematic categories based on intervention type and target population, including:

(a) interventions for ASD;
(b) interventions for LD/ID;
(c) interventions for SpLD;
(d) emerging approaches such as gamification.

### 2.6. Study Selection Summary

The study selection process is illustrated in a PRISMA flow diagram, outlining the identification, screening, eligibility, and inclusion stages.

**Figure 1:**
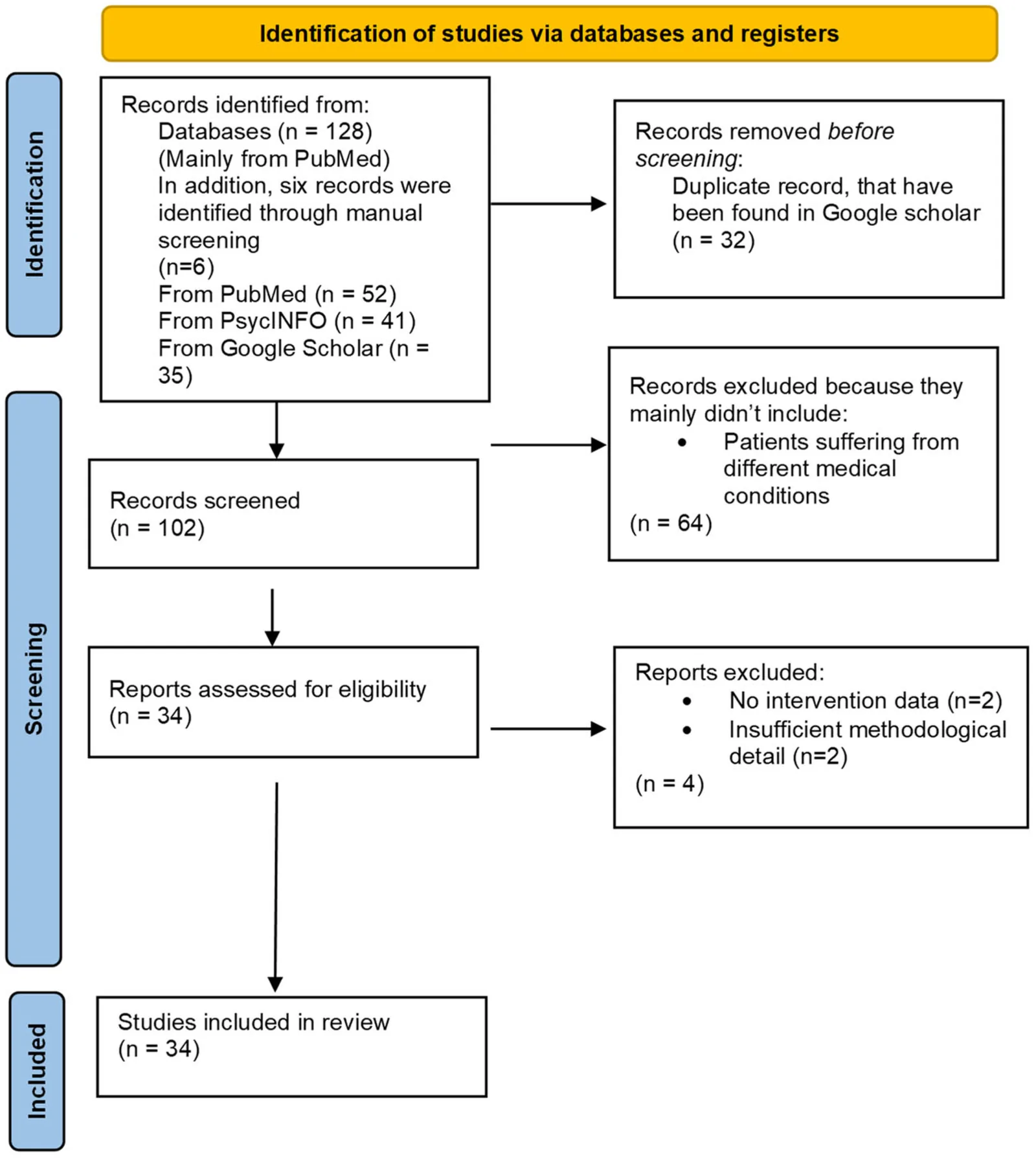
PRISMA Flow Diagram.

Note: Sample sizes (n) are approximate, as reported or derived from the included studies. The table presents a representative selection of the included studies (N = 34), illustrating the diversity of interventions, populations, and outcomes (Figure 1) (Supplementary Materials).

## 3. Results

A total of 34 studies met the inclusion criteria and were included in the final synthesis. The included studies varied in design, population, and intervention type, with a predominant focus on autism spectrum disorder (ASD), followed by learning and intellectual disabilities (LD/ID), and specific learning disabilities (SpLD).

As shown in Table 3, the included studies demonstrate substantial variability in sample characteristics, intervention types, and reported outcomes.

### 3.1. Neurodiversity-Affirming Approaches

Neurodiversity-affirming approaches emphasize the recognition of individual differences, strengths-based support, and environmental adaptation [3,14]. Across the included studies, interventions increasingly reflect a shift away from deficit-based models toward strengths-based and context-sensitive practices.

Neurodivergent individuals may demonstrate strengths such as enhanced attention to detail, creativity, and specialized interests, which can be leveraged within intervention contexts [14]. At the same time, increased vulnerability to stress, sensory sensitivities, and environmental demands highlights the importance of structured and supportive environments [44,45].

Psychoeducation and post-diagnostic support were identified as critical components of intervention, contributing to improved understanding, emotional regulation, and adaptation [46,47]. Furthermore, adaptations of therapeutic approaches—particularly in communication style and environmental structure—were found to enhance accessibility and effectiveness for neurodivergent populations [11].

Overall, the findings suggest that neurodiversity-affirming approaches are most effective when they combine individualized support, environmental adaptation, and recognition of strengths, rather than focusing exclusively on symptom reduction.

However, the degree to which the included research used strengths-based, neurodiversity-affirming methods varied significantly. Therapy activities were customized to each child’s interests, skills, developmental profile, and personal objectives, and a number of programs specifically integrated individual strengths into treatment planning. Particularly likely to emphasize child-led learning, intrinsic motivation, caregiver collaboration, and individualized support, naturalistic developmental behavioral interventions (NDBIs), person-centered therapies, and many technology-enhanced interventions respect neurodevelopmental differences while encouraging participation and functional development. These interventions promoted the growth of abilities in communication, problem-solving, creativity, social participation, and adaptive functioning and often modified therapeutic goals in accordance with children’s preexisting competencies.

On the other hand, a significant amount of traditional behavioral therapies placed very little emphasis on recognizing individual strengths and instead concentrated on lowering maladaptive behaviors or correcting developmental impairments. While many of these programs had favorable impacts on functional outcomes, there was frequently no clear mention of neurodiversity principles, self-determination, or strengths-based therapeutic planning. Although the degree of incorporation of neurodiversity-affirming techniques varied significantly between intervention types and studies, the findings generally point to an emerging movement toward customized, strengths-oriented psychological therapies.

### 3.2. Psychological Interventions for ASD

A substantial proportion of the included studies focused on interventions targeting children with ASD. Variability in age of diagnosis and access to services remains a critical issue influencing developmental outcomes [48]. Traditional approaches, particularly Applied Behavior Analysis (ABA), have demonstrated effectiveness in improving communication, social skills, and adaptive functioning [10,49]. However, concerns have been raised regarding the emphasis on behavioral normalization and potential misalignment with neurodiversity principles [12,13].

Naturalistic Developmental Behavioral Interventions (NDBI) have emerged as a more neurodiversity-aligned alternative, integrating developmental and behavioral principles within naturalistic settings [15]. These interventions emphasize child-led interaction, motivation, and the use of everyday environments, resulting in improved social communication and engagement.

Cognitive Behavioral Therapy (CBT), when adapted for autistic children, has also demonstrated effectiveness in addressing co-occurring anxiety and emotional regulation difficulties [11,17]. Additionally, autistic individuals are at increased risk for severe mental health outcomes, including suicidal ideation, highlighting the importance of early and tailored interventions [50]. Adaptations such as visual supports, structured sessions, and simplified language were identified as critical for improving accessibility.

Overall, while traditional behavioral interventions remain effective in specific domains, emerging approaches such as NDBI and adapted CBT offer greater alignment with neurodiversity principles by promoting autonomy, engagement, and contextual relevance.

An important conclusion of this analysis is that psychological therapies seem to vary based on the child’s developmental stage and the specific outcome that is being pursued. The best evidence for improving social communication, joint attention, language acquisition, play skills, and early adaptive functioning in preschoolers comes from developmental and naturalistic approaches, especially Naturalistic Developmental Behavioral Interventions (NDBIs), which capitalize on increased neuroplasticity and learning thru naturalistic interactions. During the school-age years, Applied Behavior Analysis (ABA) and associated behavioral interventions have proven effective in promoting adaptive behaviors, functional communication, and everyday life skills. Children with sufficient cognitive and verbal skills who experience anxiety, emotional dysregulation, or other co-occurring mental health problems benefit most from these interventions.

The neurodiversity framework’s tenets were reflected in these studies’ growing emphasis on meaningful participation, caregiver interaction, child interests, and tailored treatments. Nevertheless, person-centered treatment was not assessed as a separate therapeutic strategy in any of the primary studies that were included. Person-centered therapy itself was not represented in the included evidence base, despite the fact that several interventions had strengths-based or tailored components that theoretically correspond with person-centered care.

### 3.3. Interventions for Learning and Intellectual Disabilities (LD/ID)

Studies focusing on children with LD/ID indicate that psychological interventions can significantly improve academic performance, emotional well-being, and social integration [18,51]. Cognitive Behavioral Therapy (CBT) has been particularly effective in addressing anxiety, depression, and social difficulties associated with learning disabilities.

Occupational therapy and person-centered approaches were also highlighted as important interventions, supporting the development of daily living skills, self-regulation, and independence. These approaches emphasize individualized support and the active involvement of children and their families in the intervention process.

Additionally, research suggests that neurodivergent children with LD/ID are at increased risk of trauma and adverse experiences, which may negatively impact mental health outcomes [52,53]. Children with neurodevelopmental disabilities are also at increased risk of maltreatment and complex trauma, which may further exacerbate developmental challenges [54,55]. This highlights the importance of trauma-informed interventions and holistic support.

Overall, interventions for LD/ID are most effective when they adopt a holistic and individualized approach, addressing both cognitive and emotional needs while considering environmental and contextual factors.

### 3.4. Interventions for Specific Learning Disabilities (SpLD)

The literature on SpLD demonstrates a growing emphasis on strengths-based and multisensory approaches [5]. Multisensory instructional methods, incorporating visual, auditory, and kinesthetic elements, have been shown to improve engagement, memory retention, and learning outcomes [19,20].

Interventions for dyscalculia emphasize holistic approaches that address both cognitive processes and motivational factors [21,56]. Similarly, interventions for dyslexia focus on enhancing existing strengths, particularly in language and phonological processing [22]. Overall, interventions for SpLD are most effective when they move beyond deficit-focused models and incorporate multisensory, individualized, and strengths-based strategies.

### 3.5. Gamification and Digital Interventions

Emerging evidence suggests that gamification may be an effective intervention tool for neurodivergent children. Game-based approaches have been shown to improve attention, motivation, and executive functioning [23,24]. These outcomes are closely linked to broader constructs of effortful control and self-regulation, which are central to adaptive development [57].

Digital interventions, such as educational games and specialized platforms, can support cognitive development through interactive and engaging environments [25]. For example, game-based social training has been associated with improved social interaction in children with ASD [26], while digital tools such as EndeavorRx have demonstrated effectiveness in improving attention in children with ADHD [27].

However, important challenges remain, including the risk of over-reliance on digital tools and the need for careful alignment with individual needs and therapeutic goals. Overall, gamification represents a promising complementary approach, particularly when integrated with traditional and person-centered interventions. These findings provide the basis for a critical examination of the effectiveness, limitations, and theoretical implications of current intervention approaches.

## 4. Discussion

The goal of the current systematic review was to compile the available data on psychological therapies for kids with neurodevelopmental impairments using a framework that takes neurodiversity into consideration. All things considered, the results point to a significant and continuous paradigm shift away from conventional deficit-oriented models and toward more customized, strengths-based, and contextually sensitive intervention strategies. This shift is a reflection of more general advancements in modern disability and developmental studies, which place an increasing emphasis on quality of life, autonomy, and engagement in addition to traditional clinical outcomes.

Numerous studies assessed Naturalistic Developmental Behavioral therapies (NDBIs) and technology-enhanced therapies, such as gamified and digitally transmitted programs, among the recognized intervention options. The neurodiversity framework’s tenets were reflected in these studies’ growing emphasis on meaningful participation, caregiver interaction, child interests, and tailored treatments. Nevertheless, person-centered treatment was not assessed as a separate therapeutic strategy in any of the primary studies that were included. Person-centered therapy itself was not represented in the included evidence base, despite the fact that several interventions had strengths-based or tailored components that theoretically correspond with person-centered care.

Although the degree of benefit varies depending on the type of intervention and target population, the intervention studies included in this review consistently show that tailored, evidence-based approaches can improve cognitive, behavioral, emotional, and adaptive outcomes in children with neurodevelopmental disabilities. Significant improvements in language, adaptive behavior, social communication, and developmental functioning were linked to early intensive interventions, especially the Early Start Denver Model and Naturalistic Developmental Behavioral Interventions of Dawson et al., 2010, and Schreibman et al., 2015 [15,34]. Similarly, in children with autism, modified cognitive behavioral therapy successfully improved emotional regulation and decreased anxiety as suggested by Wood et al., 2009; Ozsivadjian & Knott, 2011; and Walters et al., 2016 [11,17,35]. Cognitive flexibility, attentional control, planning, and daily functioning were all improved by school- and parent-based interventions, such as executive function training and parent-mediated programs, as suggested by Kenworthy et al., 2014 [39], and Spruijt et al., 2020 [31].

Positive effects on attention, executive functioning, motivation, and social communication have also been shown by digital therapies and computer-assisted interventions like AKL-T01, Avatar Assistant, computerized working memory training, and game-based programs [24,26,27]. Additionally, children with learning disabilities and rare neurodevelopmental syndromes benefited from individualized speech-language interventions and multimodal educational approaches, which improved academic skills, communication, and functional participation [19,51]. When taken as a whole, these results support the shift away from deficit-focused therapies and toward neurodiversity-informed, strengths-based, and customized strategies that place an emphasis on quality of life, autonomy, and meaningful engagement. The requirement for high-quality randomized controlled trials assessing interventions for other neurodevelopmental conditions, such as intellectual disability, particular learning disorders, and uncommon genetic syndromes, is highlighted by the fact that the available evidence is still primarily focused on autism and ADHD.

The identification of an increasing convergence between neurodiversity-affirming recommendations and intervention techniques is a key contribution of this review. In order to enable meaningful involvement and inclusion, these methods place a high priority on acknowledging individual differences, encouraging self-determination, and modifying social and environmental circumstances [3,14]. This shift reflects broader theoretical developments that challenge strictly medicalized classifications of neurodevelopmental conditions and instead conceptualize them as part of natural human variation [1]. At the same time, emerging perspectives suggest that cognitive diversity may also contribute to innovation and adaptive functioning across social and educational contexts [4], further reinforcing the value of strengths-based approaches.

Despite the growing acceptance of a neurodiversity paradigm, the results demonstrate a continued dependence on traditional intervention strategies, especially in autism research and clinical practice. While Landa (2018) [9], Dawson et al. (2010) [34], and Peters-Scheffer et al. (2010) [38] showed that early behavioral interventions can significantly improve adaptive behavior, communication, language, and developmental outcomes in children with autism, Dixon et al. (2012) highlighted Applied Behavior Analysis (ABA) as one of the most well-known and empirically supported interventions [49]. In a similar vein, Schreibman et al. (2015) [15] recognized Naturalistic Developmental Behavioral Interventions (NDBIs) as an advancement of conventional ABA concepts, combining behavioral strategies with developmental science to foster social interaction and adaptive functioning [15]. However, ABA and comparable behavioral techniques are coming under more and more theoretical and ethical examination. While Leadbitter et al. (2021) [13] suggested that autistic viewpoints should be incorporated into intervention research and that autonomy, well-being, and quality of life should be prioritized, Chapman (2020) [12] contended that interventions that emphasize behavioral normalization may reinforce deficit-based conceptualizations of autism. Similarly, Schuck et al. (2022) [10] suggested using strengths-based, customized, and person-centered approaches to reconcile neurodiversity principles with evidence-based autism therapies. Therefore, a major problem for therapists, researchers, and policymakers continues to be striking a balance between the proven efficacy of behavioral therapies and modern ethical considerations and neurodiversity-informed values.

New intervention strategies continue to have solid empirical backing while increasingly reflecting the concepts of neurodiversity. According to Schreibman et al. (2015) [15] and Schuck et al. (2022) [10], Naturalistic Developmental Behavioral Interventions (NDBIs) emphasize children’s strengths, autonomy, and meaningful participation while integrating developmental science with naturalistic, child-led interactions to promote improvements in social communication, language, play, and adaptive functioning. Dawson et al. (2010) found that the Early Start Denver Model, an NDBI-based intervention, significantly improved young autistic children’s language development, adaptive behavior, and cognitive abilities [34]. Similarly, co-occurring emotional issues have been successfully treated with modified versions of Cognitive Behavioral Therapy (CBT). When procedures were tailored to the cognitive, social, and communicative characteristics of autistic children, Wood et al. (2009) [35], Ozsivadjian and Knott (2011) [17], and Walters et al. (2016) [11] discovered that autism-adapted CBT dramatically decreased anxiety symptoms and enhanced emotional regulation.

Furthermore, Landa (2018) highlighted that the timing of interventions is a crucial factor in determining the effectiveness of therapy, with earlier identification and availability to evidence-based treatments leading to greater increases in adaptive and developmental outcomes [9]. Mandell et al. (2005) [48] provided additional support for these conclusions by demonstrating that an earlier diagnosis is linked to better long-term developmental trajectories and enables prompt intervention. When taken as a whole, these studies highlight the significance of offering tailored, developmentally appropriate, and strengths-based therapies to children as early as feasible.

Beyond ASD, the present review highlights a relative scarcity of research focusing on children with learning and intellectual disabilities (LD/ID) and specific learning disabilities (SpLD). Although available evidence suggests that interventions such as CBT, occupational therapy, and multisensory teaching approaches can improve both academic and psychosocial outcomes [5,19,51], the evidence base remains comparatively limited and heterogeneous. Furthermore, children with neurodevelopmental disabilities are disproportionately exposed to adverse experiences, including maltreatment and trauma, which may significantly affect developmental trajectories and mental health outcomes [54,55]. These findings underscore the importance of trauma-informed and holistic intervention models.

The significance of neuropsychological and self-regulatory systems in promoting adaptive functioning is an additional significant discovery. Across neurodevelopmental problems, interventions that focus on executive functioning, attentional control, emotional regulation, and more general self-regulatory abilities seem to be especially pertinent. These abilities have been repeatedly connected to academic, social, and psychological outcomes and are intimately linked to effortful control and adaptive developmental adjustment [57]. Additionally, mental health issues such as anxiety, depression, self-harm, and suicidality are more common in neurodivergent groups [50,58,59]. Effective therapies should therefore cover more aspects of psychological well-being, resilience, and long-term quality of life in addition to symptom reduction.

By making learning and rehabilitation activities more interesting, gamification components including rewards, feedback, adaptive challenges, and interactive narratives may increase motivation and long-term involvement. Game-based approaches have demonstrated potential in improving attention, executive functioning, and social skills [23,24,26]. These interventions align well with neurodiversity principles by leveraging motivation, engagement, and individual interests. However, their long-term effectiveness and generalizability remain uncertain, and there is a need for careful consideration of potential risks, including over-reliance on digital environments and insufficient transfer of skills to real-world contexts.

The existing evidence collection has a number of limitations that should be taken into account. First, direct comparison and synthesis were hampered by the significant variation in methodological design, participant characteristics, intervention modalities, and outcome measures between studies. Second, the majority of studies on autism limit the applicability of findings to other neurodevelopmental problems, especially learning difficulties and intellectual disabilities. Third, the majority of studies focused on short-term results, with comparatively little longitudinal data looking at social involvement, quality of life, developmental trajectories, and functional outcomes over the course of a lifetime. More generally, new research shows that emotional and neurocognitive vulnerabilities often cross diagnostic borders, which supports the use of integrative and transdiagnostic methods to intervention [60,61]. Dimensional models of neurodevelopment further support these viewpoints [62].

The effectiveness of psychological therapies appears to differ depending on the child’s developmental stage and the particular result that is being targeted, which is an important conclusion of this review. By taking advantage of increased neuroplasticity and learning through naturalistic interactions, developmental and naturalistic approaches—in particular, Naturalistic Developmental Behavioral Interventions (NDBIs)—show the best evidence for enhancing social communication, joint attention, language acquisition, play skills, and early adaptive functioning in preschoolers. Applied Behavior Analysis (ABA) and related behavioral interventions have demonstrated efficacy in fostering adaptive behaviors, functional communication, and everyday living skills during the school-age years. These interventions are especially helpful for children with adequate cognitive and language abilities who suffer from anxiety, emotional dysregulation, or other co-occurring mental health issues.

Interventions for adolescents are increasingly focused on emotional well-being, self-regulation, social engagement, self-advocacy, and preparation for increased independence; person-centered therapies, cognitive behavioral therapy, family-based interventions, and technology-enhanced approaches are particularly beneficial. Technology-enhanced interventions, such as telehealth, virtual reality, digital therapeutics, and gamified platforms, provide adaptable and customized opportunities to improve executive functioning, social skills, emotional regulation, and engagement while increasing access to services across developmental stages. These results collectively imply that no single strategy is always the best; instead, the choice of intervention should be tailored to the child’s age, neurodevelopmental profile, cognitive and communicative skills, support requirements, individual strengths, and treatment objectives. The ideas of neurodiversity and personalized care, which prioritize increasing involvement, autonomy, and quality of life over achieving uniform developmental objectives, are in line with this tailored, strengths-based approach.

Finally, a critical gap in the literature concerns the limited inclusion of neurodivergent perspectives in the design and evaluation of interventions. This omission is inconsistent with the core principles of the neurodiversity movement and highlights the need for more participatory, inclusive, and ethically grounded research approaches [28,29,63]. Incorporating the voices of neurodivergent individuals is essential not only for improving intervention relevance and acceptability but also for advancing a more socially just and person-centered model of care.

### Implications and Recommendations for Practice Benefits of Different Approaches

The findings of this review suggest that practitioners should prioritize individualized, strengths-based, and context-sensitive interventions. Incorporating family involvement, environmental adaptations, and neurodivergent perspectives is essential for enhancing both effectiveness and ethical alignment of psychological interventions [30].

The advantages of various intervention strategies may differ based on the severity and features of atypical development, underscoring the significance of individualized and developmentally appropriate treatment. Structured interventions like Applied Behavior Analysis (ABA) and Naturalistic Developmental Behavioral Interventions (NDBI) may offer crucial support for children with more severe impairments in communication, adaptive functioning, or behavioral regulation by fostering foundational skills, boosting functional communication, and improving participation in everyday activities through methodical teaching and caregiver involvement. Approaches that emphasize cognitive, emotional, and social competencies—such as Cognitive Behavioral Therapy (CBT), social skills interventions, executive function training, and metacognitive strategies—may be especially helpful for kids with milder or more focused issues because they improve self-regulation, problem-solving, emotional understanding, and independence.

Additionally, occupational therapy, multisensory techniques, and environmental modifications that lower obstacles and encourage involvement may be beneficial for children with varied neurodevelopmental profiles or sensory processing impairments. By offering customized pacing, adjustable difficulty, and interesting practice opportunities, digital and gamified therapies may provide extra benefits across severity levels. Crucially, intervention objectives within a neurodiversity framework should be designed to improve strengths, autonomy, self-advocacy, and quality of life in addition to lowering functional problems. Therefore, rather than relying solely on diagnostic category or severity, the best course of action depends on the child’s particular developmental profile, support requirements, and personal objectives.

The review’s conclusions highlight the need for more thorough and methodologically sound studies assessing new psychological treatments in the context of neurodiversity. To ascertain the longevity and generalizability of treatment effects across developmental stages, future research on Naturalistic Developmental Behavioral Interventions (NDBIs) should give priority to appropriately powered randomized controlled trials, standardized intervention protocols, and long-term follow-up assessments. To promote more individualized intervention planning, more focus should be placed on identifying the child, family, and contextual variables linked to differential treatment response.

Future studies should look at person-centered treatment models as separate interventions rather than just as guiding clinical concepts, even though person-centered therapy was not directly represented among the included papers. In order to reflect outcomes that are highly valued within the neurodiversity framework, such studies should assess not only symptom reduction but also autonomy, self-determination, engagement, emotional well-being, family satisfaction, and quality of life [2].

It is also necessary to conduct further research on technology-enhanced therapies, such as telemedicine, virtual reality, digital pharmaceuticals, platforms supported by artificial intelligence, and gamified interventions. Future studies should use robust comparative designs, assess implementation in real-world contexts and long-term efficacy, and look at acceptability, cost-effectiveness, accessibility, and usability across a range of demographics. In order to make digital treatments responsive to users’ strengths, preferences, and support requirements, more work must be done to ensure that co-design and participatory approaches involving neurodivergent children, families, educators, and clinicians are used.

In general, more inclusive approaches should be used in future intervention studies by enlisting participants from a wide range of neurodevelopmental disabilities, including kids with different cognitive capacities, communication styles, linguistic and cultural backgrounds, socioeconomic situations, and co-occurring conditions. To ascertain whether early psychological therapies result in long-term gains in adaptive functioning, educational involvement, emotional well-being, independence, and general quality of life, longitudinal studies that track children into adolescence and adulthood are especially necessary. When taken as a whole, these developments would improve the body of knowledge and make it easier to create tailored, neurodiversity-affirming interventions that encourage meaningful engagement throughout life.

## 5. Conclusions

Using the neurodiversity framework as a lens, this systematic study illustrates how psychological therapies for children with neurodevelopmental disorders are changing. All things considered, the data points to a gradual transition away from traditional deficit-based models and toward customized, strengths-oriented methods that acknowledge neurodivergent traits as normal variances in human development rather than impairments that need to be normalized. Newer intervention models, such as Naturalistic Developmental Behavioral Interventions (NDBIs), parent-mediated programs, executive function training, multisensory educational approaches, digital therapeutics, and gamified interventions, are increasingly consistent with neurodiversity-affirming principles, even though well-established interventions like Applied Behavior Analysis (ABA) and adapted Cognitive Behavioral Therapy (CBT) continue to show strong evidence for improving adaptive functioning, communication, and emotional regulation as Dawson et al., 2010; Wood et al., 2009; and Walters et al., 2016 suggested [11,34,35].

The assessment found a number of significant gaps in the current body of research, despite these positive advancements. While much fewer high-quality studies examine interventions for children with intellectual disabilities, specific learning disorders, developmental coordination disorder, or uncommon neurodevelopmental syndromes, the majority of intervention studies still focus primarily on autism spectrum disorder. Furthermore, awareness of the long-term impact of therapies on quality of life, independence, academic achievement, and community participation is limited by the very small number of randomized controlled studies that include long-term follow-up. The inadequate inclusion of neurodivergent perspectives in the creation and assessment of interventions is another significant drawback. Future research should include co-production and participatory approaches that actively incorporate neurodivergent people and their families, in accordance with the suggestions made by Chapman (2020) [12] and Leadbitter et al. (2021) [13]. By ensuring that therapeutic success is assessed not only through symptom reduction but also through well-being, self-determination, social inclusion, and overall quality of life, such interventions are likely to improve the ecological validity, acceptability, and person-centeredness of interventions.

The review further reveals important gaps in the literature. The evidence base remains heavily centered on autism, with comparatively limited research addressing learning and intellectual disabilities and specific learning disabilities. Furthermore, there is a lack of longitudinal studies examining long-term outcomes, particularly in relation to quality of life, autonomy, and social inclusion. The limited incorporation of neurodivergent perspectives in intervention design further highlights the need for more inclusive and participatory research approaches [13].

Future research should prioritize the development and evaluation of interventions that are not only evidence-based but also ethically grounded and responsive to the lived experiences of neurodivergent individuals [31]. Cross-diagnostic approaches, interdisciplinary collaboration, and the integration of families and communities are essential for advancing both research and practice [64]. Ultimately, psychological interventions are most effective when they move beyond symptom reduction and support meaningful participation, well-being, and quality of life for neurodivergent children [32]. These findings have important implications for clinical practice, policy development, and inclusive education systems [65].

## Figures and Tables

**Table 1:** Summary of the electronic database search strategies used in the systematic review.

Database	Search Strategy (Summary)	Filters/Limits
**PubMed**	Combined terms related to neurodiversity/neurodivergence, psychological and behavioral interventions (e.g., CBT, ABA, NDBI, psychotherapy, treatment), children/adolescents, and neurodevelopmental disorders (ASD, ADHD, LD/ID, SpLD, dyslexia, dyscalculia).	English language; peer-reviewed studies; publication years 2010–2025.
**PsycINFO**	Combined terms for neurodiversity, psychological/behavioral interventions (including CBT, ABA, NDBI, game-based and digital interventions), children/adolescents, and neurodevelopmental disorders (ASD, ADHD, LD/ID, SpLD, dyslexia, dyscalculia).	English language; peer-reviewed journal articles; publication years 2010–2025.
**Google Scholar**	Combined keywords related to neurodiversity, psychological and behavioral interventions (CBT, ABA, NDBI, digital interventions, gamification), children/adolescents, and neurodevelopmental disorders (ASD, ADHD, learning disabilities, intellectual disability, SpLD, dyslexia).	English-language publications; publication years 2010–2025.

**Table 2:** Methodological details of the present systematic review.

Methodological Issues	Details
**Inclusion Criteria**	Children (0–18 years) with ASD, LD/ID, or SpLD; psychological, behavioral, or educational interventions; cognitive, behavioral, emotional, or QoL outcomes; English-language, peer-reviewed studies.
**Exclusion Criteria**	Adult-only studies; no intervention; theoretical/opinion papers; conference abstracts, editorials, and non-peer-reviewed publications.
**Study Selection Process**	Stage 1: Screening of titles and abstracts for relevance. Stage 2: Full-text assessment against eligibility criteria. Screening was performed independently by the authors, and disagreements were resolved through discussion and consensus.
**Data Extraction Framework**	Authors, publication year, participant characteristics, intervention type, duration/setting, and cognitive, behavioral, emotional, and psychosocial outcomes.

**Table 3:** Characteristics of included studies.

Author (Year)	Sample	Diagnosis	Intervention Type	Duration	Key Outcomes
Axbey, et al. (2023) [4]	Early intervention research and practice	71 participants autistic-only, non-autistic-only, and neurodiverse pairs	Neurodiversity-informed early intervention principles	Varied	Neurodiverse pairs would generate more innovative (less similar) solutions than single-neurotype pairs
Sewell, (2022) [5]	Children with Specific Learning Difficulties (narrative synthesis)	SpLD (including dyslexia)	Neurodiversity-informed educational and psychological supports	Varied across studies	Strengths-based educational practices improved inclusion, learner engagement, and educational participation.
Landa (2018) [9]	Infants and young children with ASD or with high likelihood of ASD (reviewed studies)	ASD	Early developmental and behavioral interventions (e.g., ESDM, parent-mediated interventions, ABA-based programs)	Varied across studies	Improved social communication, language, adaptive functioning, and developmental outcomes, particularly with early intervention.
Schuck et al. (2022) [10]	Children (*n* ≈ 80; school-aged)	ASD	Behavioral interventions (reviewed approaches)	Variable	Improved adaptive and social functioning
Walters et al. (2016) [11]	Children & adolescents (*n* ≈ 60; ages 8–16)	ASD	Adapted Cognitive Behavioral Therapy (CBT)	8–16 weeks	Reduced anxiety symptoms
Schreibman et al. (2015) [15]	Children (*n* ≈ 120; ages 2–8)	ASD	Naturalistic Developmental Behavioral Intervention (NDBI)	12–24 weeks	Improved social communication and engagement
Toki, et al. (2024) [16]	Children with communication and swallowing disorders (systematic review)	Communication and swallowing disorders	Technology-assisted interventions (mobile applications, digital tools, AAC, assistive technologies)	Varied across studies	Digital technologies improved communication, engagement, and therapeutic accessibility.
Ozsivadjian & Knott (2011) [17]	Children (*n* ≈ 20; ages 8–14)	ASD	Cognitive Behavioral Therapy (CBT)	10–12 weeks	Improved anxiety management
Mehtar & Mukaddes (2011) [18]	Children (*n* ≈ 30; ages 6–16)	ASD	Cognitive Behavioral Therapy (CBT)	10 weeks	Reduced trauma-related symptoms
Abdulkarim et al. (2017) [19]	Students (*n* ≈ 50; primary education)	SpLD	Multisensory intervention	8 weeks	Improved motor and writing skills
Newman (2018) [20]	Students (*n* ≈ 40; secondary education)	SpLD	Multisensory teaching strategies	Variable	Increased engagement and retention
Kucian & von Aster (2014) [21]	Children (*n* ≈ 70; ages 7–12)	SpLD (dyscalculia)	Educational intervention	Variable	Improved mathematical skills
Yu et al. (2018) [22]	Children (*n* ≈ 60; school-aged)	SpLD (dyslexia)	Language-based intervention	Variable	Enhanced reading and language abilities
Diamond & Lee (2011) [23]	Children (*n* ≈ 100; ages 4–12)	Developmental delays	Cognitive training interventions	Variable	Improved executive functioning
Prins et al. (2011) [24]	Children (*n* ≈ 50; ages 8–12)	ADHD	Game-based cognitive training	5 weeks	Improved attention and impulse control
Kebritchi et al. (2010) [25]	Students (*n* ≈ 193; middle school)	LD	Game-based learning	6–8 weeks	Improved academic performance and motivation
Hopkins et al. (2011) [26]	Children (*n* ≈ 49; ages 8–12)	ASD	Game-based social skills training	10 weeks	Improved social interaction
Kollins et al. (2020) [27]	Children (*n* ≈ 348; ages 8–12)	ADHD	Digital therapeutic intervention (EndeavorRx)	4 weeks	Improved sustained attention
Fuller & Kaiser (2020) [28]	Young children (*n* ≈ 200; ages 1–5)	ASD	Early intervention (meta-analytic synthesis)	Variable	Improved social communication outcomes
Gregory & Nichols (2018) [29]	Children (*n* ≈ 80; school-aged)	Mixed neurodevelopmental profiles	Environmental/trauma-informed school-based intervention	Variable	Improved behavioral regulation and school adaptation
Papadopoulou et al. (2025) [30]	Children (*n* ≈ 25; ages 3–10)	Rare neurodevelopmental disorders	Speech and language therapy	Variable	Improved communication and feeding skills
Spruijt et al. (2020) [31]	Children (*n* ≈ 70; school-aged)	Neurodevelopmental disorders	Parent-mediated intervention	Variable	Improved attentional control and parent–child interaction
Sauer et al., (2025) [32]	Toddlers and preschool children with autism	ASD and developmental disabilities	Evaluates an NDBI	Varied	Social communication, autism symptoms, developmental functioning through evidence-based behavioral interventions.
Ma, & Song, (2025) [33]	Children with ASD	ASD	Technology-enhanced (VR and serious games)	16 weeks	Improved social communication, emotional regulation, learning, and rehabilitation-related skills
Dawson et al. (2010) [34]	48 toddlers (18–30 months)	ASD	Early Start Denver Model (ESDM)	2 years	Significant improvements in IQ, adaptive behavior, language, and reduction in autism symptom severity compared with community treatment.
Wood et al. (2009) [35]	40 children (7–11 years)	High-functioning ASD with anxiety	Adapted Cognitive Behavioral Therapy (CBT)	16 sessions	Anxiety symptoms decreased significantly; treatment gains maintained at 3-month follow-up.
Kasari et al. (2012) [36]	69 school-aged children	ASD	NETT (Nonverbal communication, Emotion recognition, Theory of Mind Training) social skills program	12 sessions	Improved social communication, empathy, and peer relationships compared with active control.
Tamm et al. (2010) [37]	23 children	ADHD	Pay Attention! attention training program	Up to 16 sessions	Improved attention, executive functioning, working memory, and reduced ADHD symptoms.
Scheffer et al. (2010) [38]	34 preschool children	ASD with intellectual disability	Low-intensity ABA preschool intervention	8 months	Greater gains in adaptive behavior and developmental functioning than standard preschool services.
Kenworthy, et al. (2014) [39]	67 children (Grades 3–5)	Autism Spectrum Disorder (ASD)	Unstuck and On Target (UOT)—school-based executive function intervention	Approximately 6 months (school-based program)	Greater improvements in cognitive flexibility, planning, problem-solving, classroom behavior, and transitions than social skills training.
de Vries, et al. (2015) [40]	121 children (8–12 years)	Autism Spectrum Disorder (ASD)	Computerized working memory and cognitive flexibility training (Braingame Brian)	25 training sessions (≈5–6 weeks)	Improved working memory, cognitive flexibility, attention, and parent-rated executive functioning, although differences between intervention conditions were modest.
López-Nieto et al. (2022) [41]	71 children	Autism Spectrum Disorder (ASD)	Play-based, peer-mediated pragmatic language intervention	10 weekly sessions	Significant improvements in pragmatic language skills, peer interaction, and social communication.
Faja, et al. (2022) [42]	70 children (7–11 years)	Autism Spectrum Disorder (ASD)	Web-based executive function training with metacognitive coaching	10 weeks	Improved executive functioning and reduced restricted and repetitive behaviors
Wood, et al. (2020) [43]	107 children (6–13 years)	Autism Spectrum Disorder (ASD)	Modular Cognitive Behavioral Therapy (CBT)	32 therapy sessions	Improved social engagement and reduced autism-related social symptoms compared with enhanced community treatment.

## Data Availability

No new data were created or analyzed in this study. Data sharing is not applicable to this article.

## Supplementary Materials

The following supporting information can be downloaded at https://www.mdpi.com/article/10.3390/children13081071/s1. PRISMA 2020 Checklist.
